# Repurposing of Antibiotics: Sense or Non-sense

**DOI:** 10.3389/fphar.2022.833005

**Published:** 2022-02-21

**Authors:** Absar Talat, Yasir Bashir, Asad U. Khan

**Affiliations:** ^1^ Interdisciplinary Biotechnology Unit, Aligarh Muslim University, Aligarh, India

**Keywords:** NDM-4, antibiotic resistance, proteomics, carbapenem resistance, *K. pneumoniae*

## Introduction

Drug repurposing (also called drug repositioning, re-profiling or re-tasking) is a strategy for identifying new uses for approved or investigational drugs that are outside the scope of the original medical indication (Ashburn and Thor, 2004). It has gained a lot of attention in recent years. The number of research projects for utilising already available drugs against different diseases has gained momentum in current times. The development of a new drug demands exorbitant funds, human resources and unpredictable amount of time (Ventola, 2015). Several studies have shown promising results of repurposed drugs for combating different diseases (Zhang et al., 2020). Currently, the scientific journals have been flooded with the utility of repurposed drugs especially in the times of COVID-19 when the shortage of drugs threatened the whole world. Drug repurposing has been employed in order to speed up the traditional process of drug discovery by bypassing the need for toxicity testing for drugs that have already been proven to be safe and effective in humans and approved by the FDA (Food and Drug Administration, United States) for other indications (Pushpakom et al., 2019).

One potential aspect that has been overlooked while drug repurposing is Antimicrobial Resistance (AMR). It has become the most dreaded menace increasing at an alarming rate and threatening the global health. The emergence of antimicrobial resistant bacteria is one of the most important public health challenges worldwide (World Health Organization, 2014). World Health Organization (WHO) lists the overuse and misuse of antibiotics to be the major cause of AMR (https://www.who.int/campaigns/world-antimicrobial-awareness-week/2021). Recent studies have shown the simmering public health crisis with increase in resistance against antibiotics of last resort, carbapenems and polymixins (Gandra et al., 2016). In 2019, drug-resistant infections across 88 pathogen-drug combinations caused 4.95 million deaths globally, out of which 1.27 million deaths were directly linked to AMR (Murray et al., 2022). So, there is an urgent need to address the issues related to the overuse and misuse of antibiotics and save the humankind from another global pandemic.

## Discussion

**FIGURE 1:**
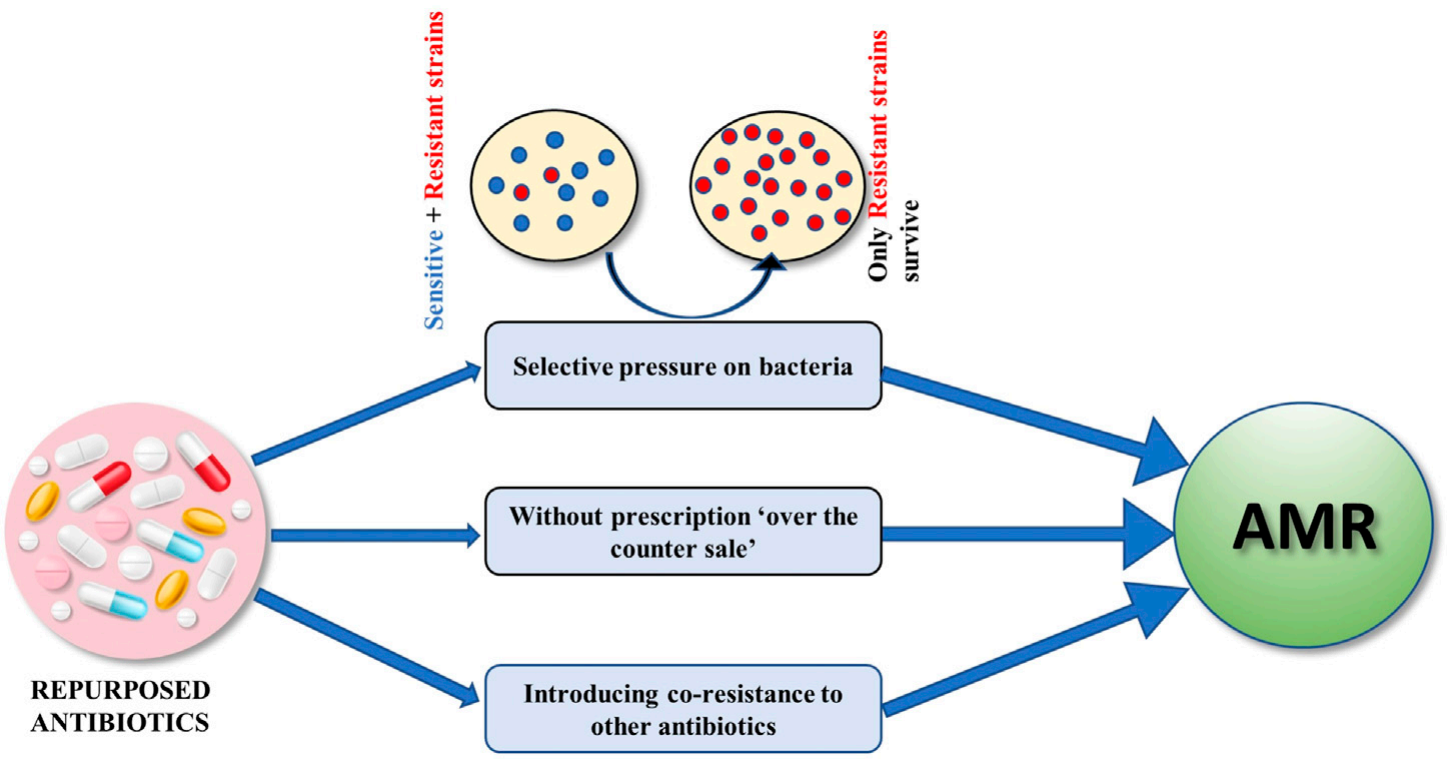
Repurposed antibiotics can lead to an increase in AMR by exerting selective pressure killing off sensitive strains, uncontrolled without prescription consumption as well as by causing co-resistance to other antibiotics.

While the concept of drug repurposing sounds logical and a scientific way of dealing with hitherto untreatable diseases viz. Alzheimer’s, Cancer, Parkinson’s etc., the repurposing of antibiotic drugs has raised eyebrows over the use of antibiotics in treating such diseases given the rampant antimicrobial resistance that has wreaked havoc in contemporary times. AMR has been known to cause over 700,000 deaths globally and is estimated to kill around 10 million people worldwide by 2050 (O'Neill, 2014). There are many studies that have used antibiotics in order to treat diseases other than the bacterial infections and tried to repurpose antibiotics (Song et al., 2016; Alsalahat et al., 2021; Khan et al., 2021) but these studies have overlooked the grave danger of using antibiotics for repurposing, i.e, AMR progression (Figure 1).

The antibiotic levofloxacin has been recently suggested as a potential repurposed drug for Alzheimer’s disease (Khan et al., 2021) while several other studies showing the repercussions of using this antibiotic in Alzheimer’s patients have been neglected. One of them being a case report of increased seizures in Alzheimer’s disease (AD) patient when administered with 500 mg/day dose of levofloxacin (Bird et al., 2005). Also, the gut microbiome of Alzheimer’s patients has shown the role of *H. pylori* infection in AD patients as triggers for the release of inflammatory mediators (Doulberis et al., 2021). *H. pylori* is known for its resistance against levofloxacin (Mehrotra et al., 2021). The major concern of any repurposed antibiotic is its dosage, duration for which it is supposed to be consumed and its consequences on AMR. Antibiotics are mostly administered at doses much higher than non-antibiotic drugs (Farha and Brown, 2019). At a high concentration of 400 mg, minocycline was found to be in-effective in delaying the progress of cognitive or functional impairment in patients with mild AD during a 2 year period (Howard et al., 2020). Though such antibiotic drugs prove to be potential candidates *in vitro*, their efficacy is not warranted *in vivo*. Further, the long-term exposure to an antibiotic either at clinical or sub-stoichiometric dose can induce drug resistance among the gut microbes. There is a need to administer these antibiotics chronically for the treatment or management of AD. Therefore, chronic use of antibiotics at clinical dose may impose antibiotic resistance as a result of which it would become difficult to treat the common infection. Additionally, the long-term use of antibiotics may make the patients more prone to infection. If any antibiotics are showing anti-AD potential either *in vitro* or clinical studies, then, long-term consequences of using these antibiotics need to be evaluated. There has been no substantial study to understand the long-term effects of using repurposed antibiotics on enhancing AMR but as prior data show a direct correlation between antibiotic consumption and AMR (Gandra et al., 2016), there may be an escalation of multi-drug resistance with increasing consumption of these repurposed antibiotics. For instance, doxycycline is a repurposed antibiotic used for treating malaria. Though there are no reports about an increase in AMR because of doxycycline in malaria prophylaxis but there are reports of the doxycycline resistance and co-resistance against other antibiotics while treating other diseases. A study showed that doxycycline stress resulted in co-resistance to colistin, a last-resort antibiotic, to treat extensively drug-resistant bacteria (Narendrakumar et al., 2020). The study illustrated a possible mechanism of doxycycline-selected resistance and co-resistance in *V. cholerae* and warranted strict restrictions on the indiscriminate use of antibiotics. Another facet is the limitation of unrestricted sale of these antibiotics without prescription especially in developing countries like India, one of the largest antibiotic consumer (Kotwani et al., 2021). So, the controlled usage of these repurposed antibiotics will be jeopardized. The long-term usage of the repurposed antibiotics may exert the selective pressure leading to the survival of multi-drug resistant and extremely drug resistant bacteria. In the absence of a competitive environment, these drug resistance strains may thrive much better with the killing off of phenotypically sensitive strains (Fair and Tor, 2014).

During the COVID-19 pandemic, the health practitioners resorted to the prescription of azithromycin alone as well as in combination with drugs like hydroxychloroquine. It became a highly controversial repurposing of an antibiotic without any proof of its antiviral properties. However, its use still amplified with successive waves of COVID-19. Only time will tell as to what will be the ramifications of such an unregulated and unscientific use of such antibiotics. Its consequences on AMR will worsen the situation (Yacouba et al., 2021). Given the positive association between heavy antibiotic use and worsening of antibiotic resistance crisis in the current times, efforts to strengthen AMR stewardship must be made at an international level so as to reduce the impact of antibiotic use on the menace of antibiotic resistance and emphasis must be given to understand the consequences of antibiotic repurposing.

There is utmost need to find alternatives of antibiotics in repurposing, especially those which have been already shown to cause resistance. The successful repurposing of non-antibiotic drugs against cancer has been reported (Advani and Kumar, 2021; Tímár et al., 2005) and the same approach can be adopted for other diseases as well. The repurposed antibiotic surpasses the clinical trials but in absence of concrete studies addressing the grave situation of AMR, it may turn out to be a catastrophe for the already progressing AMR against most of the antibiotics. The role of AMR in aggravating the problems in treating nosocomial infections and mortalities due to multi-drug resistance can’t be undermined, especially in the times when most of the antibiotics are failing and the treatment regimen involves last-resort antibiotics (Avershina et al., 2021). We must find a way to not exert further burden on the already existing AMR load. Hence, we suggest the clinicians and researchers to raise awareness about the rampant AMR and the consequences thereof and discourage the use of antibiotics in drug repurposing.
